# Plant-Based Alternatives to Cheese Formulated Using Blends of Zein and Chickpea Protein Ingredients

**DOI:** 10.3390/foods12071492

**Published:** 2023-04-01

**Authors:** Nadia Grasso, Francesca Bot, Yrjo H. Roos, Shane V. Crowley, Elke K. Arendt, James A. O’Mahony

**Affiliations:** 1 School of Food and Nutritional Sciences, University College Cork, T12 TP07 Cork, Ireland; 2 Department of Food and Drug, University of Parma, 43124 Parma, Italy

**Keywords:** plant protein ingredients, plant-based cheese, microstructure, meltability, stretching, texture

## Abstract

In this study, zein protein isolate (ZPI) and chickpea protein concentrate (CPC) ingredients were used to formulate five plant-based cheese alternatives. Ingredient ratios based on protein contributions of 0:100, 25:75, 50:50, 75:25 and 100:0 from ZPI and CPC, respectively, were used. Formulations were developed at pH ~4.5, with a moisture target of 59%. Shea butter was used to target 15% fat, while tapioca starch was added to target the same carbohydrate content for all samples. Microstructural analysis showed differences among samples, with samples containing ZPI displaying a protein-rich layer surrounding the fat globules. Schreiber meltability and dynamic low amplitude oscillatory shear rheological analyses showed that increasing the proportion of ZPI was associated with increasing meltability and greater ability to flow at high temperatures. In addition, the sample containing only CPC showed the highest adhesiveness, springiness and cohesiveness values from the texture profile analysis, while the sample containing only ZPI exhibited the highest hardness. Furthermore, stretchability increased with increasing ZPI proportions. This work will help understanding of the role and potential of promising plant-protein-ingredient blends in formulating plant-based alternatives to cheese with desirable functional properties.

## 1. Introduction

Cheese represents an important food product in many cultures, with a long history of production and consumption [1]. Dairy production sectors have rapidly intensified over the past several decades, leading to high productivity, which, although increasing overall economic profits has been accompanied by some undesirable social and environmental consequences [2]. On the other hand, the availability of new food products is expanding, leading consumers to consider numerous factors when purchasing their food [3]. In particular, drivers known to enhance consumer interest in plant-based food include food intolerances, social trends, and environmental sustainability, health and animal welfare considerations [4,5]. As a result, the plant-based cheese- alternative sector is growing, and household penetration of this category is on the rise as more consumers experiment with plant-based cheese. Indeed, in the US between 2020 and 2021, the percentage of households purchasing plant-based cheese increased by 20% [6].

Plant-protein ingredients are currently being studied for their potential in the development of plant-based alternatives to cheese. However, designing plant-based products with composition and functionality that closely match the properties of traditional cheese is challenging [5]. Indeed, dairy proteins are largely responsible for the unique nutritional, physicochemical and sensorial attributes of cheese products. At present, commercially available plant-based cheese alternatives rely strongly on non-protein ingredients (i.e., starch and coconut oil) to deliver functionality to the final product, resulting in low protein and high saturated fat contents. Moreover, the composition, microstructure and functional properties (e.g., meltability) of such products differ considerably from those of cheese [7]. Plant-protein ingredients can be employed in the development of new and reformulated foods with improved nutritional profiles, while also providing specific desirable functional attributes [8].

Among the plant-protein ingredients studied for application in alternative cheese products, pulses are considered to be a valuable source of macronutrients, with functionality of relevance in such applications [9]. In particular, chickpeas are very nutritious and represent an important source of dietary protein, with a content of 20–25%, as well as having high contents of fat, starch and fibre, minerals and vitamins [10]. Furthermore, due to the predominance of globulin, chickpea protein ingredients show good oil absorption capacity and emulsification and gelling properties [11,12,13]. Chickpea protein ingredients have been studied by our group in relation to the development of plant-based cheese alternatives, and have shown both promising results and challenges, the latter mainly related to poor meltability of the final product [14]. Besides pulses, another plant protein that displays strong potential in plant-based cheese alternative applications is zein, due to its unique plastic behaviour in aqueous environments [15]. Zein is the hydrophobic major prolamin fraction extracted from maize, in which it represents 45–50% of the total protein [16]; it is composed of α, β, γ and δ fractions and is soluble in aqueous ethanol solutions. However, the production process for commercial zein results primarily in the α fraction [17]. Due to its structure and high proportion of non-polar amino acids, zein is self-aggregating and hydrophobic, and non-covalent interactions are largely responsible for its ability to form viscoelastic networks [18,19]. Given these unique physicochemical characteristics, zein has been extensively used to entrap other substances, such as vitamins, and to stabilise oil-in-water emulsions [20]. From a nutritional point of view, based on the Protein Digestibility-Corrected Amino Acid Score (PDCAAS), zein can be compared to wheat proteins, which are usually combined with complementary proteins to improve their nutritional value without losing functionality [21]. 

The use of blends of different plant proteins In food products is increasing (e.g., in commercial plant-based milk alternatives), providing improvements in the physicochemical, nutritional and sensory properties of the final product [22,23]. In this study, zein and chickpea protein ingredients were formulated in binary blends and their combined effect on the development and physicochemical properties of plant-based cheese alternatives was investigated. The results of this work will help in furthering understanding of the role and potential of blends of promising plant-protein ingredients in the formulation of plant-based alternatives to cheese with desirable functional properties.

## 2. Materials and Methods

### 2.1. Ingredients 

The cheese alternative samples were formulated using commercially available zein protein isolate (ZPI) (Flo Chemical Corporation, Ashburnham, MA, USA), with 81.5% protein, 5.62% moisture, 3.92% carbohydrate, 7.68% fat and 1.28% ash, and chickpea protein concentrate (CPC) (Artesa, PLT Health Solutions, Morristown, NJ, USA), with 53.1% protein, 7.73% moisture, 33.3% carbohydrate, 1.37% fat and 4.47% ash. Tapioca starch, with 0.11% protein, 11.3% moisture, 88.3% carbohydrate, 0.25% fat and 0.03% ash, was purchased from a local retail outlet (Quay-coop, Cork, Ireland). Shea butter was kindly provided by Fuji Oil (Zenitex M 50 G, Fuji Oil Europe, Ghent, Belgium), and was employed as a source of solid fat and as an alternative to the more commonly used coconut oil, which has a higher saturated-fat content. Sunflower lecithin powder (Bungemaxx^®^) was obtained from Bunge-Loders Croklaan (Rotterdam, The Netherlands).

### 2.2. Formulation of Cheese Alternative Samples 

Blends of CPC and ZPI were used to formulate the cheese alternatives (Table 1). Increasing proportions of ZPI were used to obtain five different formulations, 0Z-100C, 25Z-75C, 50Z-50C, 75Z-25C and 100Z-0C, with ingredient ratios designed according to protein contributions of 0:100, 25:75, 50:50, 75:25 and 100:0 from ZPI and CPC, respectively. Shea butter was included to achieve a fat content of 15%, and a 2.3 M lactic acid solution was mixed with the water to obtain a pH of ~4.5 and 59% moisture for all the samples. Moreover, tapioca starch was added at increasing concentrations, from 0.10 to 10.6% (Table 1), to target a carbohydrate content of 10.1% for all samples. Samples were prepared as described by Grasso et al. [14], with slight differences. In brief, the powder ingredients were mixed with water (previously combined with the lactic acid solution) in a Thermomix (TM 5, Vorwerk, Wuppertal, Germany) at speed 1 (100 rpm) for 1 min; then, the temperature was set to 100 °C; and finally, when a temperature of 40 °C was reached (after ~30 s), shea butter was added at speed 2.5 (350 rpm) for 5.5 min. After heating, samples were transferred into moulds and analysed after 24 h of storage at 4 °C.

### 2.3. Compositional and Colour Analyses of Powder Ingredients and Cheese Alternatives

Composition of the ZPI and tapioca starch was analysed, while composition of the CPC ingredient was previously determined by Grasso et al. [14]. Total nitrogen content of the ingredients and cheese alternative samples was measured using Kjeldahl methods 930.29 [24] and 2001.14 [25], respectively, with a nitrogen-to-protein conversion factor of 6.25. Moisture content of the ingredients and cheese alternative samples was determined by oven drying, according to method 925.10 [26] and method 926.08 [27], respectively. Ash content was determined by dry ashing in a muffle furnace according to method 923.03 [28] for the ingredients and method 935.42 [29] for the cheese alternative samples. Fat content was measured according to the Soxhlet AACC method 30–25.01 [30]. Total carbohydrate was calculated by difference (i.e., 100 minus the sum of protein, fat, ash and moisture). The pH, water activity (a_w_) and colour of the samples were measured as previously described by Grasso et al. [14].

### 2.4. Protein Profile Analysis of Protein Ingredients 

Protein profile of CPC was previously assessed by Grasso et al. [14], and protein profile of ZPI was measured following the same method, using a capillary electrophoresis instrument (PA 800 plus Pharmaceutical Analysis System, Sciex, Kildare, Ireland).

### 2.5. Differential Scanning Calorimetry Analysis of the Ingredients and Cheese Alternatives

Thermograms of the tapioca starch and cheese alternative samples were obtained using a Mettler DSC821 (Mettler-Toledo, Schwerzenbach, Switzerland) differential scanning calorimeter (DSC). Shea butter and CPC thermograms were previously measured by Grasso et al. [14], while the tapioca starch was weighed (5.5–7.1 mg) into standard aluminium pans and water (~10 mg) was added to hydrate the powder. The cheese alternatives were weighed (2.8–4.9 mg) into aluminium pans which were hermetically sealed. The thermal behaviour of the ingredients and cheese alternative samples was assessed using the method reported by Grasso et al. [14].

### 2.6. Confocal Laser Scanning Microscopy

Microstructural analysis of the cheese alternatives was carried out using an OLYMPUS FV1000 confocal laser scanning biological microscope (Olympus Corporation, Japan). Fat and protein of samples were stained as previously described by Le Tohic et al. [31]. Nile red and fast green FCF were excited at 488 and 633 nm, using Ar and He-Ne lasers, respectively [32]. Images of the cheese alternative samples, obtained using a 40× objective lens, were reported. Images of the 100% zein sample obtained using a 60× objective lens were also reported.

### 2.7. Dynamic Low Amplitude Oscillatory Shear Rheology

Rheological properties of the products were measured using an AR-G2 controlled-stress rheometer (TA Instruments Ltd., Waters LLC, Leatherhead, UK) equipped with crosshatched-surface stainless-steel parallel plates. Samples were prepared and analysed according to the method described by Grasso et al. [7]. The exposed edges of the cheese alternative samples were coated with liquid paraffin to prevent drying. The viscoelastic behaviour of the system is characterised by the storage (G′) and loss (G″) moduli, while the ratios between the two moduli is defined as the loss tangent (tan *δ*) [33]. The melting index was calculated from G′ at 20 and 80 °C according to the following Equation (1):

(1) $$\mathrm{Melting}\;\mathrm{index}\;(\%)=\frac{{G^{\prime }}_{20}-{G^{\prime }}_{80}}{{G^{\prime }}_{20}}\cdot 100$$

### 2.8. Schreiber Meltability Test

Meltability of the cheese alternatives was assessed using the Schreiber test as previously described by Altan et al. [34]. Samples were prepared as described by Grasso et al. [14] and heated at 232 °C for 5 min in an oven (Memmert, Schwabach, Germany). After 30 min at room temperature, specimen expansion was measured using a ruler along six lines marked on a series of concentric circles. Meltability was taken as the mean of the six readings and expressed as percentage sample expansion [35]. Photographs of the samples were taken before and after heating.

### 2.9. Extensibility Analysis

A texture analyser TA-XT2i (Stable Micro Systems, Godalming, Surrey, UK) with a cheese extensibility rig (model A/CE) attachment equipped with a PT100 temperature probe was used to assess the extensibility of the samples. The cheese alternative samples were weighed (60 g) and distributed evenly on the fork in the sample pot. The sample pot was heated for 12 min in the oven (Memmert, Schwabach, Germany) at 220 °C. The sample-pot assembly was then inserted into the slotted base, and the PT100 probe was placed into the cheese. Once the temperature reached 55 °C the test started, pulling the fork out of the melted sample. The test speed and distance were set to 10 mm/s and 220 mm, respectively. From the raw data obtained from the Exponent Connect (version 8,0,7,0) computer software, the area under the curve from 0 to 10 s was calculated for all samples. 

### 2.10. Texture Profile Analysis 

Texture profile analysis (TPA) was performed using a texture analyser TA-XT2i (Stable Micro Systems, Godalming, Surrey, UK), using the method previously described by Grasso et al. [14], with the cheese alternative samples being stored at 4 °C for 24 h in the moulds before being removed and analysed. Textural parameters, hardness, adhesiveness, springiness and cohesiveness were measured.

### 2.11. Statistical Data Analysis

Compositional analysis of the ingredients, and of the cheese alternative samples, was performed in triplicate, as well as DSC analysis of the ingredients. Protein profile analysis of ZPI was performed in duplicate. Three independent trials were conducted to develop the cheese alternative samples, and three independent replicates from each trial were used for all analyses. The homogeneity of variance was assessed using Levene’s test, and one-way analysis of variance (ANOVA) was performed using SPSS version 25 (SPSS Inc., Chicago, IL, USA). A Tukey’s paired-comparison post hoc test was carried out to identify significant differences (*p* < 0.05) between mean values of samples, at a 95% confidence level.

## 3. Results and Discussion

### 3.1. Composition of Powder Ingredients and Cheese Alternatives 

#### 3.1.1. Chemical Composition

Composition of the ingredients was used to formulate the plant-based cheese alternative samples (Table 1). As expected from the formulations, the samples had similar macronutrient contents; importantly, protein content was not significantly different among the plant-based cheese alternatives, and the same was observed for moisture and pH (Table 2). Furthermore, slight differences were observed in the fat contents of the cheese alternatives, with a target value of 15% for all formulations. To achieve a pH of ~4.5, increasing amounts of lactic acid were used with increasing CPC content (Table 1), as previously observed by Grasso et al. [14], probably because of the buffering capacity of the globulin fractions of chickpea protein [36]. The a_w_ of the cheese alternatives ranged between 0.985 and 0.997.

#### 3.1.2. Colour 

**Figure 1:**
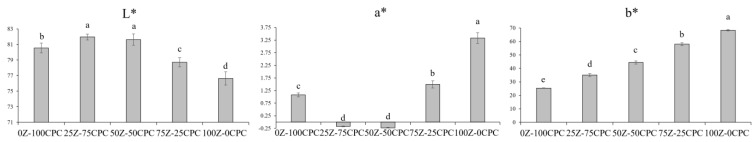
Colour space values (L* a* b*) of plant-based cheese alternative samples. Different letters (a–e) indicate significant differences between samples (*p* < 0.05).

The CIELAB coordinate values of the cheese alternatives are reported in Figure 1. The b* coordinate values indicate the degree of yellowness (or blueness for negative values), and these values increased with the ZPI addition level, showing the highest value for the 100Z-0C sample, due to the characteristic strong yellow colour of ZPI. This value was higher also when compared with commercially available plant-based and dairy cheeses previously studied, which ranged between 26.2 and 46.1 [7]. Moreover, the a* coordinate values, with the colour green representing negative values and the colour red representing positive values, were the highest for the sample containing no CPC (towards the colour red) and negative for the 25Z-75C and 50Z-50C samples. The opposite trend was observed for the L* coordinate (which represents the lightness), where the lowest value was observed for the 100Z-0C sample; this value was lower than commercially available plant-based and dairy cheeses [7].

#### 3.1.3. Protein Profile of Powder Ingredients

The protein profile of ZPI under non-reducing and reducing conditions is reported in Supplementary Figure S1. As expected, the electropherogram showed the typical zein profile, with two main peaks at 19 and 22 kDa corresponding to α-zein, the major fraction found in commercial zein, as previously reported [17,18,37,38]. Moreover, smaller peaks around ~48 kDa were evident, in particular under non-reducing conditions. These might correspond to a dimer of γ-zein, which is also visible from the lower MW peak around 18 kDa [17]. Esen [39] proposed the classification of zein fractions into α-, γ-, β- and δ-zein, which correspond to ~71–85%, 10–20%, 1–5% and 1–5%, respectively, of total protein [17,40]. The protein profile of CPC as previously studied showed mainly the 11S legumin and 7S vicilin globulin fractions [14], which represent 53–60% of total protein in chickpeas [41]. Under reducing conditions, the CPC electropherogram showed peaks around 35–40 and 20 kDa, corresponding to the 11S legumin acidic (α-legumin) and basic (β-legumin) chains, respectively. Subunits of 7S vicilin corresponded to the peaks around 50 kDa (i.e., major fraction) and around 15, 32 and 70 kDa (i.e., minor subunits) of the CPC electropherograms.

### 3.2. Thermal Behaviour of the Powder Ingredients and Cheese Alternatives

**Figure 2:**
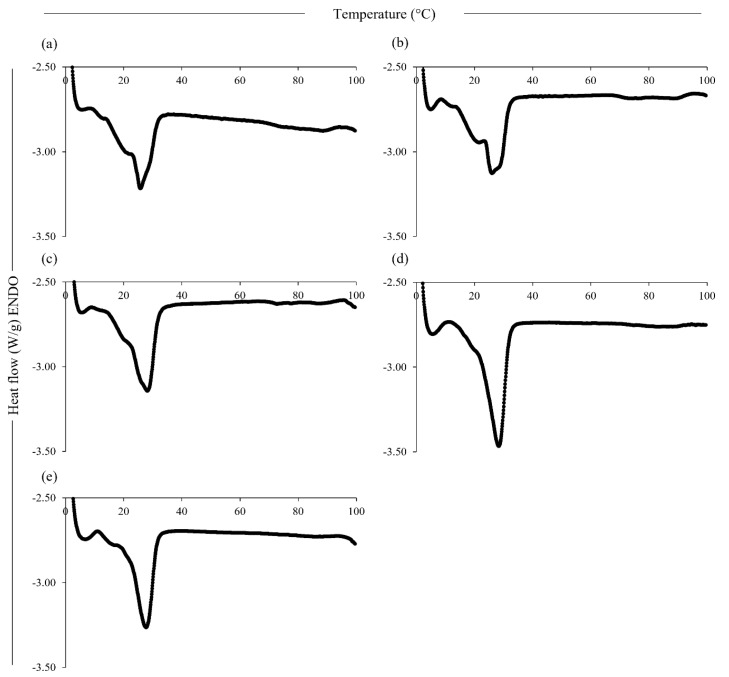
Differential scanning calorimetry thermograms of the plant-based cheese alternative samples, 0Z-100C (**a**), 25Z-75C (**b**), 50Z-50C (**c**), 75Z-25C (**d**) and 100Z-0C (**e**).

The DSC thermograms of the cheese alternatives are reported in Figure 2. A main peak was observed for all samples around 30 °C, corresponding to melting of the shea butter [14]. However, the samples with higher ZPI contents showed a narrow peak shape, probably linked to the differences in structure of the samples and the distribution of fat and protein within them. Two small peaks around 75 °C and 90 °C were shown by the thermograms of the samples containing CPC, corresponding to the denaturation of the globulin fractions, in agreement with the thermogram of the CPC ingredient, which previously displayed a main peak at 93.7 °C and a smaller peak at 77.1 °C, corresponding to the denaturation of the (7S) vicilin and (11S) legumin fractions, respectively [14].

The gelatinisation of the tapioca starch occurred at 61.4 °C (thermogram not reported), in agreement with values reported in the literature [42]. However, the transition of the tapioca starch, used in the formulations in different proportions, was not visible from the thermograms of the samples; an explanation for this might be the reduction in the gelatinisation enthalpy due to the effect of the ZPI, as previously observed for mixtures of proso millet starch and zein [43]. This decrease might be attributed to the competition between starch and protein for available water, or due to the distribution of the zein on the surface of the starch granules, which protects the structure and prevents swelling, similar to the behaviour reported for mixtures of *α*-casein and waxy maize starch [44]. Furthermore, the denaturation of the zein was not visible from the sample thermograms, with the denaturation temperature of dry zein powder reported in the literature to be ~139 °C; however, zein is significantly plasticised by water, and transition temperatures can vary according to the a_w_ of zein [45]. 

### 3.3. Microstructure

**Figure 3:**
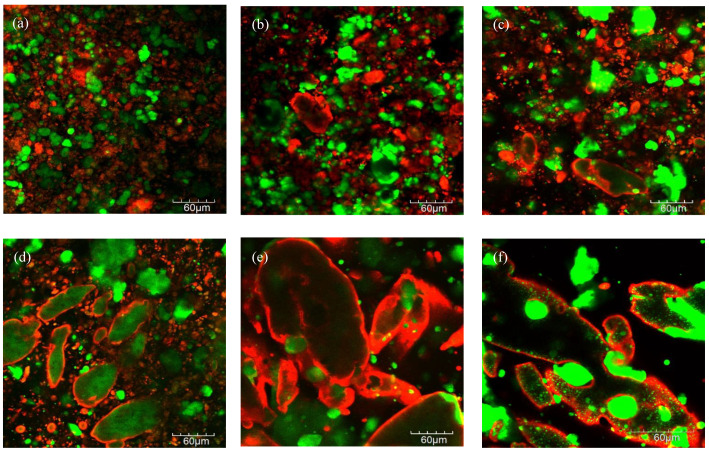
Confocal laser scanning microscopy images of the plant-based cheese alternative samples, 0Z-100C (**a**), 25Z-75C (**b**), 50Z-50C (**c**), 75Z-25C (**d**) and 100Z-0C (**e**), at 40×. Microstructure of 100Z-100C (**f**) at 60× is also shown. Fat and protein are represented by green and red, respectively.

Analysis of the microstructural images showed considerable differences between samples (Figure 3). Samples containing ZPI (Figure 3b–f) showed evidence of a protein layer surrounding the fat globules, while the chickpea proteins were more homogeneously distributed. The hydrophobicity of zein, and, therefore, its ability to orientate at the interface of fat droplets, has been previously used to stabilise Pickering emulsions [46]. Pools of fat globules were observed for all samples; however, the size of these pools increased with increasing ZPI content, with the largest non-spherical coalesced pools of fat globules evident in the 100Z-0C sample (Figure 3e,f). Some aggregation of chickpea protein was observed in all samples containing the CPC ingredient (Figure 3a–d). This was in agreement with a previous study, in which the aggregation of pea proteins was observed in formulations of zein and pea-protein dough [47]. The black areas in the images represent carbohydrate, which was present at the same concentration for all samples. These differences in microstructure are reflected in the differences in physicochemical properties between the cheese alternative samples. 

### 3.4. Rheological Properties

**Figure 4:**
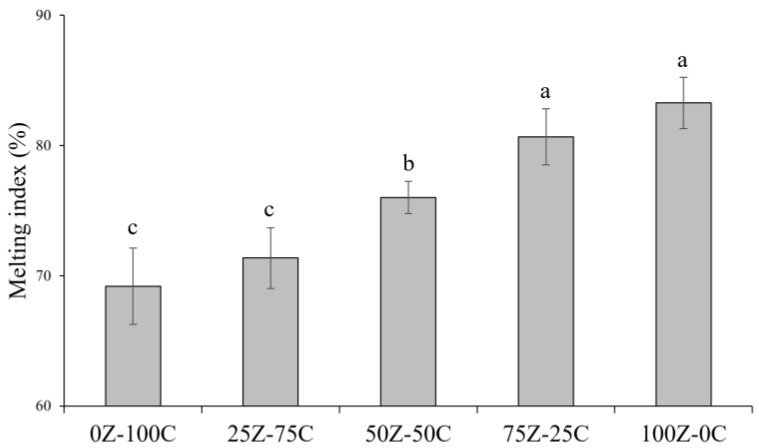
Meltability index of plant-based cheese alternative samples. Different letters (a–c) indicate significant differences between samples (*p* < 0.05).

The rheological profiles and the melting index of the cheese alternative samples are reported in Supplementary Figure S2 and Figure 4, respectively. The rheological behaviour of cheese on heating is linked to shrinkage of the *para*-casein network, which occurs between 60 and 90 °C, and the consequent expulsion of moisture, leading to complete transformation from viscoelastic solid to liquid [33]. As shown in Figure 4, increased proportions of ZPI resulted in more extensive melting behaviour of the cheese alternatives. Particularly at high temperatures, the increasing melting index, as well as the decreasing distance between G′ and G″ (Figure S2), with increasing ZPI content, indicated greater viscous behaviour and ability to flow at high temperatures for samples with higher ZPI contents compared with samples having lower ZPI contents. This behaviour was in agreement with previous observations on zein-based products [15] and is likely to be linked to the weakening of non-covalent bonds in zein at high temperatures, which is strongly responsible for its viscoelastic behaviour [19]. From 20 to 35 °C, all samples showed softening, with rapid decreases in G′ values; this is likely to be due to melting of the shea butter, which had a transition temperature of 35 °C (Section 3.2). Furthermore, tan ẟ increased between 60 and 80 °C with increasing ZPI content, suggesting increasing ability of the samples to flow with increasing proportions of zein, with only the 100Z-0C sample having a final tan ẟ value higher than the respective initial value. 

### 3.5. Meltability

**Figure 5:**
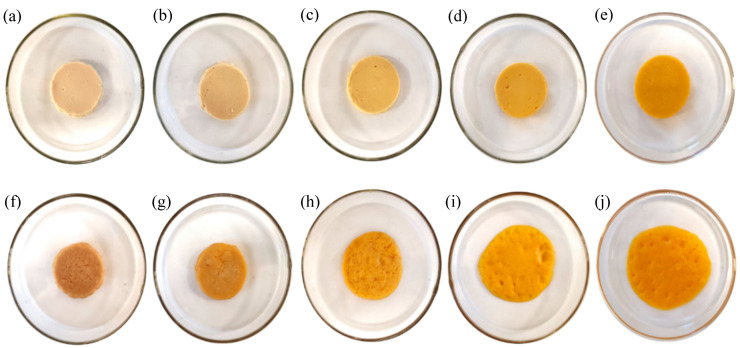
Photographs of plant-based cheese alternative samples, 0Z-100C, 25Z-75C, 50Z-50C, 75Z-25C and 100Z-0C before (**a**–**e**) and after (**f**–**j**) oven heating at 232 °C for 5 min.

**Figure 6:**
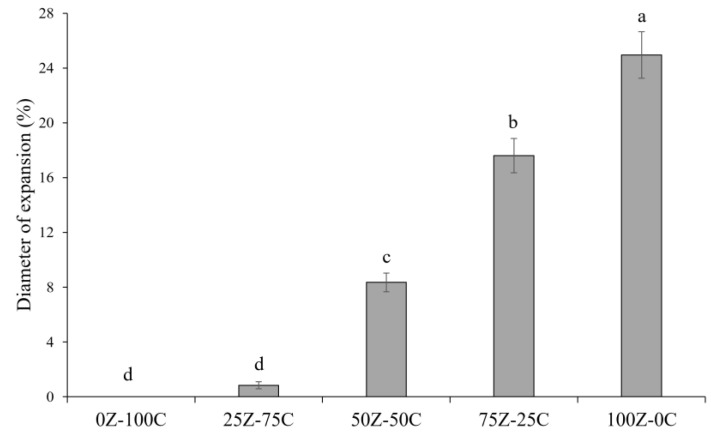
Schreiber meltability test of plant-based cheese alternative samples. Different letters (a–d) indicate significant differences between samples (*p* < 0.05).

Meltability has previously been defined as the ease with which cheese flows or spreads upon heating [48] and is a key quality attribute of cheese products. The photographs of the samples before and after heating and the data for the meltability of the cheese alternatives in this study are reported in Figure 5 and Figure 6, respectively. Increasing proportions of ZPI were associated with increasing meltability, in agreement with the rheological data (Section 3.4). This was evident from the photos of the samples before and after heating (Figure 5), with the 100Z-0C sample showing the highest meltability, with 25% diameter expansion (Figure 6). This sample displayed better meltability than commercial plant-based cheese alternatives previously studied; in addition, the 75Z-25C sample showed greater extent of diameter expansion (17.6%) than most such products [7]. As for the rheology results, at high temperatures, samples containing zein showed ability to flow, probably due to weakening of non-covalent bonds in zein, similar to casein networks in traditional cheese, as previously observed by Mattice and Marangoni [15,21]. Indeed, due to its water insolubility, when hydrated and heated above its glass transition temperature, zein self-assembles in a plastic and viscoelastic mass [21]. The 0Z-100C did not show any diameter expansion, with similar results reported for the 25Z-75C sample; indeed this limited meltability of chickpea-based cheese alternatives was previously reported [14].

### 3.6. Stretchability

**Figure 7:**
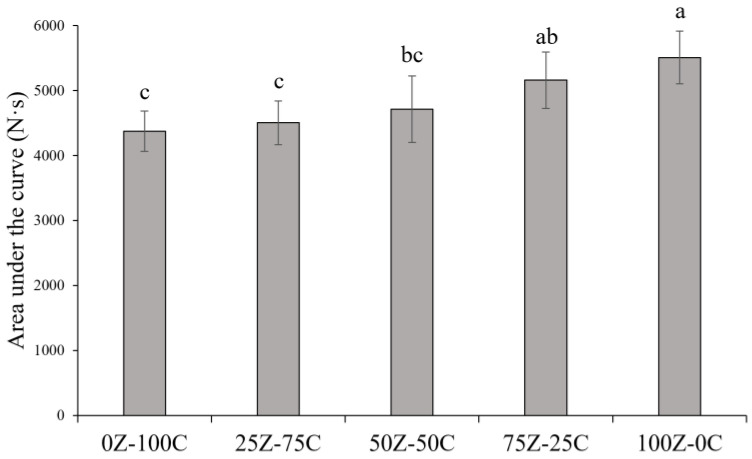
Area under the force–time curve from time 0 to 10 s of plant-based cheese alternative samples. Different letters (a–c) indicate significant differences between samples (*p* < 0.05).

**Figure 8:**
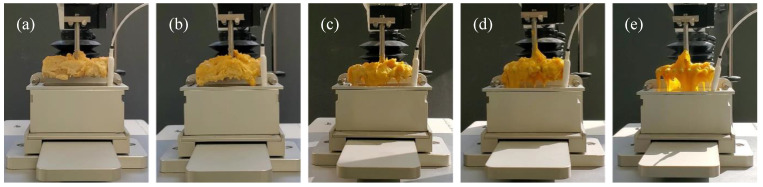
Photographs of melted plant-based cheese alternative samples, 0Z-100C (**a**), 25Z-75C (**b**), 50Z-50C (**c**), 75Z-25C (**d**) and 100Z-0C (**e**) during stretchability test.

The stretchability of melted cheese represents an important quality attribute. In this study, the area under the force–time curve in the interval 0–10 s was calculated and used as a parameter to compare stretching of the samples. As reported in Figure 7, increasing proportions of ZPI were associated with increasing area values and, thus, increased stretching, as is also evident from the photographs of the samples (Figure 8). The sample containing only ZPI showed the highest area value, statistically comparable only to the 75Z-25C sample. Samples with high CPC contents maintained a rigid structure and did not stretch under the testing conditions; as reported in Section 3.5, such samples were unable to achieve a molten mass—a prerequisite for subsequent stretching of the sample. In cheese, stretching is defined as the ability of the casein network to maintain its integrity, without breaking, when a continuous stress is applied [49]. Similarly, due to weakening of non-covalent interactions, zein networks soften at high temperatures, leading to enhanced stretching properties, as previously observed by Mattice and Marangoni [21]. 

### 3.7. Textural Properties 

**Figure 9:**
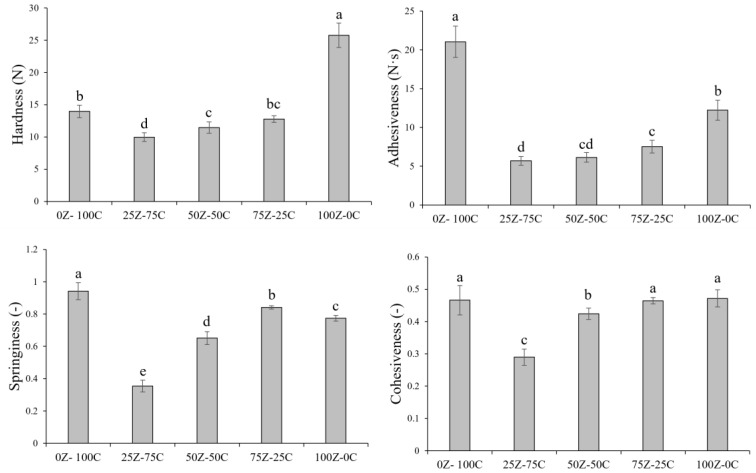
Texture profile analysis parameters, hardness, adhesiveness, springiness and cohesiveness of plant-based cheese alternative samples. Different letters (a–e) indicate significant differences between samples (*p* < 0.05).

The texture parameters of plant-based cheese alternative samples are shown in Figure 9. Hardness, defined as the height of the force peak on the first compression cycle [50], was the highest for the 100Z-0C sample, significantly different from the other samples. Interestingly, the samples containing both ZPI and CPC had lower hardness than the ZPI-only and CPC-only samples. The 25Z-75C sample had the lowest values for all parameters, with the other two blends (i.e., 50Z-50C and 75Z-25C) showing higher values. These differences are likely to be linked to the interactions between the two protein ingredients (i.e., ZPI and CPC) and the structure of these samples, as previously reported in Section 3.3. The 0Z-100C sample showed the highest adhesiveness, springiness and cohesiveness values, with the latter being statistically comparable to the 100Z-0C and 75Z-25C samples. Adhesiveness is correlated with the interactions between fat and protein, which influence the adherence between the product and the contact surface, as well as the structure of the protein matrix [51]. The 0Z-100C sample had a protein matrix which differed considerably to that of the samples containing ZPI, which may have been responsible for the significantly higher adhesiveness of this sample. Moreover, the different starch (i.e., chickpea starch vs tapioca starch) and the overall carbohydrate profiles of the samples are likely to have influenced texture; the final carbohydrate content (i.e., ~10.1%) was constant for all samples.

## 4. Conclusions

The effect of different ratios of ZPI and CPC on the development and physicochemical properties of plant-based cheese alternatives was investigated. Large differences were observed in the microstructure of the cheese alternative samples, with ZPI forming a protein layer surrounding the fat globules. Improvements in the meltability and stretching behaviour of the samples were associated with increasing ZPI content, due to its viscous and plastic nature in aqueous environments and the ability of zein to flow at high temperatures. Furthermore, samples showed different texture, with the 100Z-0C sample having the highest hardness value and the 0Z-100C sample the highest adhesiveness, springiness and cohesiveness. The results of this work show the potential of blending plant protein ingredients in the development of promising plant-based alternatives to cheese with desirable functional properties and assist in our understanding of their role in formulating such products.

## Figures and Tables

**Table 1:** Formulation (%) of the cheese alternative samples.

	ZPI	CPC	Starch	Shea Butter	Lecithin	Lactic Acid	Water
0Z-100C	0	29.9	0.10	14.4	0.20	8.50	48.2
25Z-75C	4.90	22.5	2.80	14.1	0.20	6.30	50.4
50Z-50C	9.80	15.0	5.40	13.8	0.20	4.30	52.4
75Z-25C	14.6	7.50	8.00	13.6	0.20	2.10	54.6
100Z-0C	19.5	0	10.6	13.3	0.20	0.10	56.6

ZPI = zein protein isolate, CPC = chickpea protein concentrate.

**Table 2:** Composition of plant-based cheese alternative samples.

	Protein (%)	Moisture (%)	Carbohydrate (%)	Fat (%)	Ash (%)	pH (-)	a_w_ (-)
0Z-100C	16.3 ± 0.26 ^a^	56.2 ± 0.01 ^a^	11.1	15.3 ± 0.33 ^ab^	1.40 ± 0.04 ^a^	4.55 ± 0.01 ^a^	0.985 ± 0.000 ^e^
25Z-75C	16.2 ± 0.17 ^a^	56.8 ± 0.52 ^a^	10.9	15.0 ± 0.13 ^b^	1.01 ± 0.01 ^b^	4.54 ± 0.02 ^a^	0.988 ± 0.001 ^d^
50Z-50C	16.1 ± 0.06 ^a^	56.7 ± 0.04 ^a^	11.4	15.0 ± 0.37 ^b^	0.73 ± 0.03 ^c^	4.55 ± 0.01 ^a^	0.992 ± 0.002 ^c^
75Z-25C	16.3 ± 0.17 ^a^	56.3 ± 0.20 ^a^	11.6	15.5 ± 0.11 ^ab^	0.49 ± 0.02 ^d^	4.53 ± 0.02 ^a^	0.994 ± 0.001 ^b^
100Z-0C	16.3 ± 0.30 ^a^	56.2 ± 0.34 ^a^	10.7	15.9 ± 0.34 ^a^	0.22 ± 0.01 ^e^	4.53 ± 0.01 ^a^	0.997 ± 0.001 ^a^

Values followed by different superscript letters (a–e) in a column are significantly different (*p* < 0.05).

## Data Availability

Data are presented within the article or Supplementary Materials.

## Supplementary Materials

The following supporting information can be downloaded at https://www.mdpi.com/article/10.3390/foods12071492/s1: Figure S1: Protein profile of zein protein isolate (ZPI) under non-reducing and reducing conditions. The bottom electropherogram represents the molecular weight (MW) standard. Figure S2: Rheological profiles showing storage modulus (G′) (open triangle), loss modulus (G″) (open square) and loss tangent (tan δ) (filled circle) as a function of temperature in the range 20–80 °C for 0Z-100C (a), 25Z-75C (b), 50Z-50C (c), 75Z-25C (d) and 100Z-100C (e) plant-based cheese alternative samples.
