# Supplementary material for: Plant-Based Alternatives to Cheese Formulated Using Blends of Zein and Chickpea Protein Ingredients
Source: Foods. 2023 Apr 1;12(7):1492. doi: 10.3390/foods12071492 (PMC10093979; doi:10.3390/foods12071492)
Supplement: Supplementary file 1 [file foods-12-01492-s001.zip › foods-2291845-supplementary.pdf]

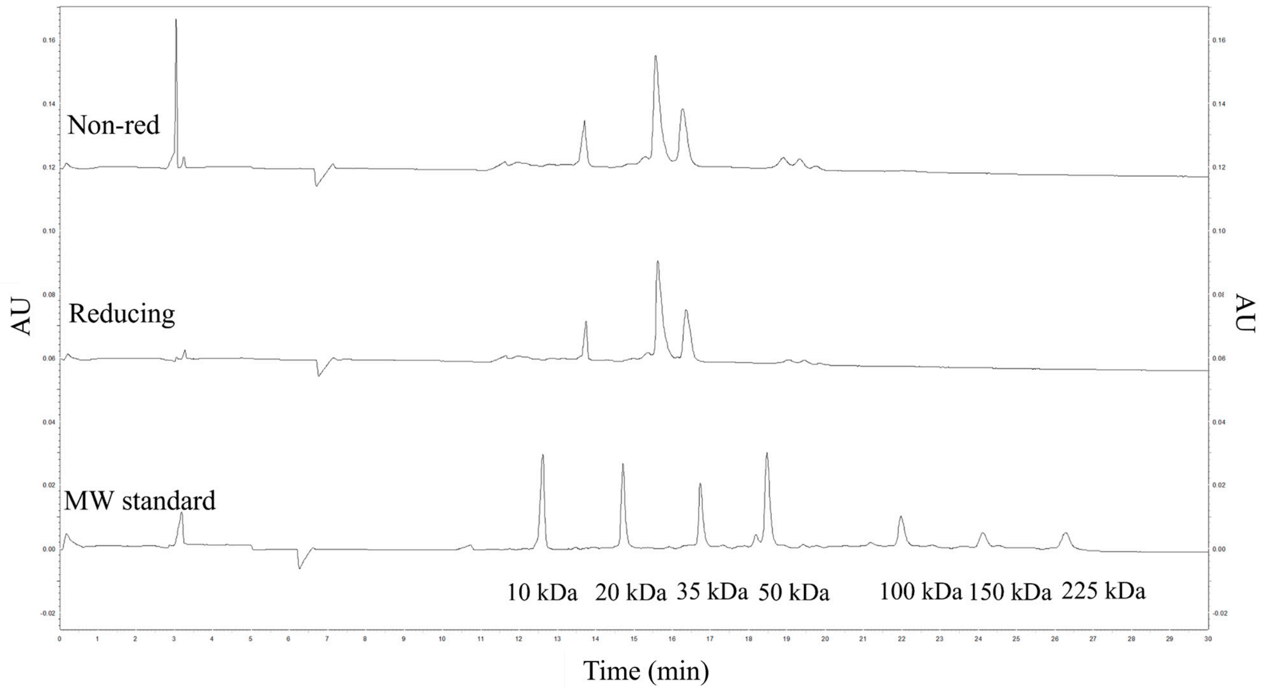

Figure S1. Protein profile of zein protein isolate (ZPI) under non reducing and reducing conditions. The bottom electropherogram represents the molecular weight (MW) standard.

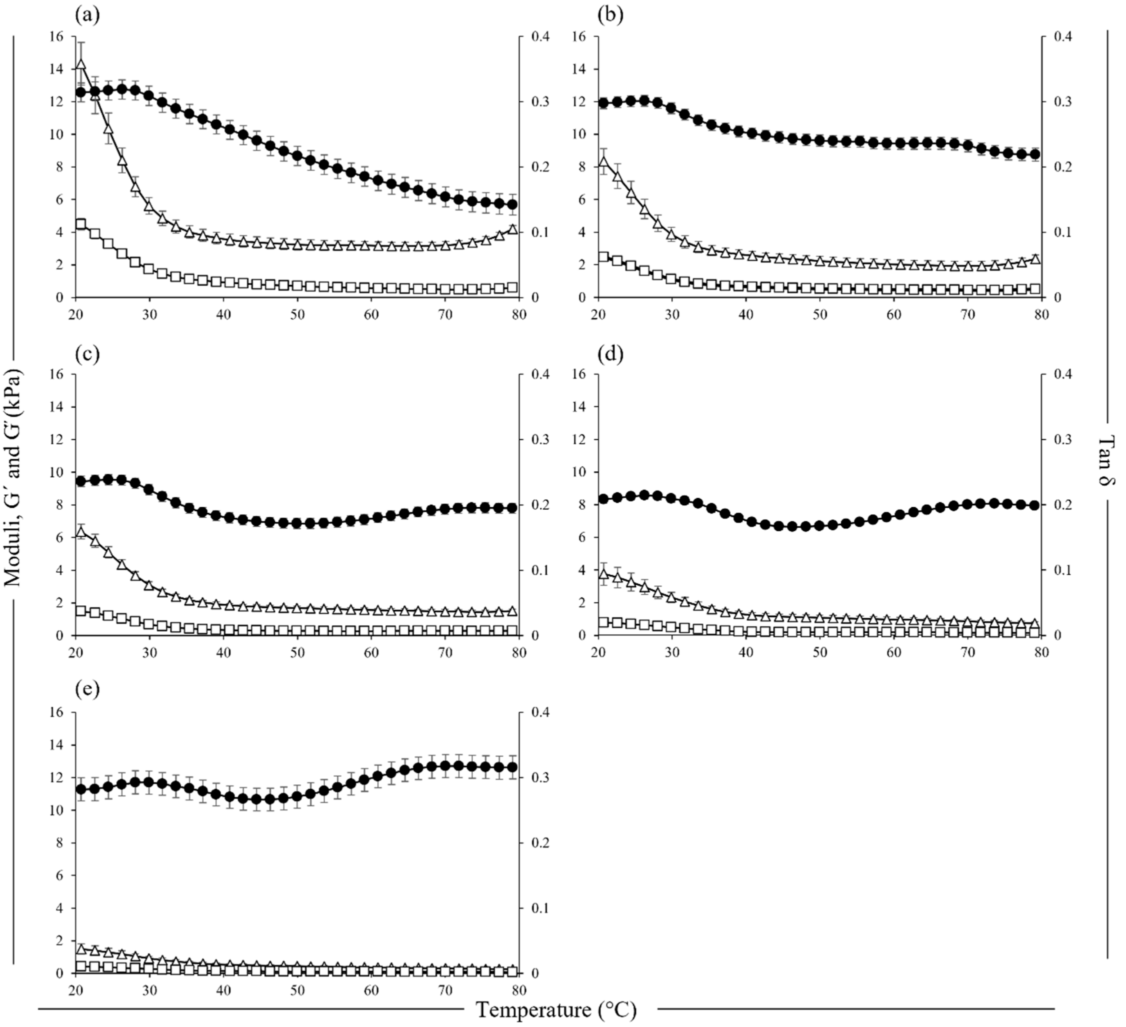

Figure S2. Rheological profiles showing storage modulus (G′) (open triangle), loss modulus (G″) (open square) and loss tangent (Tan *δ*) (filled circle) as a function of temperature in the range 20–80°C for 0Z-100C (a), 25Z-75C (b), 50Z-50C (c), 75Z-25C (d) and 100Z-100C (e) plant-based cheese alternative samples.
